# Measurement of Factors Affecting the Perception of People with Disabilities in the Workplace

**DOI:** 10.3390/ijerph17124455

**Published:** 2020-06-21

**Authors:** Urszula Załuska, Alicja Grześkowiak, Cyprian Kozyra, Dorota Kwiatkowska-Ciotucha

**Affiliations:** 1 Department of Logistics, Wroclaw University of Economics and Business, 53-345 Wrocław, Poland; urszula.zaluska@ue.wroc.pl; 2 Department of Econometrics and Operational Research, Wroclaw University of Economics and Business, 53-345 Wrocław, Poland; alicja.grzeskowiak@ue.wroc.pl; 3 Department of Statistics, Wroclaw University of Economics and Business, 53-345 Wrocław, Poland; cyprian.kozyra@ue.wroc.pl

**Keywords:** disability, WHO ADS scale, inclusive employment, confirmatory factor analysis, structural equations modelling

## Abstract

The issue of employing people with disabilities is crucial from both a social and economic perspective, and is often influenced by the social perception of this group of people. In this article, we attempted to examine attitudes towards the disabled in eight European countries by using one of the most popular tools that measures the perception of such people in everyday life—the Attitudes to Disability Scale (ADS) developed by the WHOQOL Group. We checked the general attitude towards disability according to the ADS scale and the specific perception of disability in the workplace using a scale created ad hoc. The research was conducted in 2019 using the CAWI (computer-assisted web interview) method on representative samples of Internet users, whereas the analysis methods included the measurement reliability analysis, confirmatory factor analysis (CFA) and structural equation modelling (SEM). The obtained results allow for the acceptance of the measurement model of the ADS scale in the societies of the analyzed countries. No significant differences were found between models created for people with a disability experience (a group from the WHOQOL Group research) and without such experience. The measurement using the original ADS scale factor structure is of good reliability, whereas CFA is of good fit. We also examined the impact of ADS scale factors on the perception of people with disabilities in the workplace using the SEM model, and obtained good fit of the model. The results show that the dimensions of perception, such as inclusion, discrimination and prospects, affect the evaluation of people with disabilities in the workplace.

## 1. Introduction

One of the key elements of sustainable economic development is ensuring that all people function normally in society, and have equal access to employment. Although several years have passed since the Convention on the Rights of Persons with Disabilities [1] was enacted by the United Nations, the employment rate for this group of people is still much lower in most European and world countries when compared with social demographics [2,3]. Inequalities also relate to access to healthcare [4] or disproportions in the level of future retirement benefits [5], in particular for people with disabilities from older age groups [6]. The results of many scientific studies indicate that one of the reasons for this may be poor knowledge of the specificity of different types of disability in societies, and as a result, an unfavourable social perception of such people. Obtaining remuneration for one’s work instead of allowance is more beneficial for both the economy and employees. However, disability stereotypes may cause additional difficulties in finding employment. The literature on the subject includes many examples of research dedicated to, among others, attitudes towards people with disabilities and ways of measuring them [7,8]. In the review of research on employers’ attitudes towards people with disabilities [9], which included 34 studies from 1987–2012, there were three distinguished groups of factors affecting employment: adaptation of people with disabilities in the workplace, work efficiency, and reactions of colleagues as well as employers’ attitudes. Actions undertaken in practice indicate much lower openness to people with disabilities than declared [10]. Many research results seem to confirm that it is the attitude of employers and able-bodied colleagues that is of decisive importance for the success of the adaptation process of people with disabilities in the workplace [11,12]. The negative attitudes of employers constitute the main barrier to employing people with disabilities, which has been confirmed by, among others, research conducted in Sweden [13,14].

For many years, a lot of time has been devoted to developing and improving tools for measuring the perception of people with disabilities in everyday life. What was important in this regard was the work on the development of the Attitudes to Disability Scale (ADS) test [15,16,17] performed by a team led by M. Power (The World Health Organization Quality of Life Group—WHOQOL Group). The ADS test was created as an auxiliary measure in developing an intercultural tool for measuring the quality of life of people with physical or intellectual disabilities (WHOQOL-DIS). The ADS questionnaire asked respondents about their opinion on disability and disabled people in general. The test consists of sixteen statements related to the perception of the functioning of people with disabilities in society in four main areas (factors): inclusion (items 1–4), discrimination (items 5–8), gains (items 9–12), prospects (items 13–16)—see Table 1. Respondents express their opinions using the 5-point Likert scale, where 1 means “I completely disagree”, and 5 “I completely agree”. The following stages of developing the ADS test were verified in 14 research centres around the world, and the target group included people who experienced disability in some way-people who suffered from disabilities and those who took care of them (e.g., caregivers, therapists). The authors of the scale obtained quite good results in terms of reliability of measurement, although there were noted significant differences depending on the centres where the study was conducted, gender or type of disability. Classical psychometric analyses (based on linear correlations) and those performed with the use of IRT (item response theory) methodology based on the logit analysis made it possible to obtain convergent results in both methods for the same data. The items of the ADS scale presented in Table 1 are formulated as negative statements about people with disabilities. The exception is factor 3 (gains), in which items indicate positive attributes related to disability.

The results of the research obtained using the ADS WHOQOL Group scale have inspired us to design an international survey on representative samples of respondents from eight European countries. The first objective of the survey was to check whether the ADS scale can be used to measure the attitudes of the whole society, and not only of people with disability experience, i.e., people suffering from disabilities and people directly dealing with them (e.g., caregivers, physiotherapists, people from institutions helping the disabled). We also strived to examine whether the same measurement tools can be used in groups of people who experienced disability (as in the work of the WHOQOL Group) and groups of people without such experience. The results confirming this assumption would mean that the representation of the societies of the analyzed countries can be included in the sample while performing analyses. That would be a valuable result extending the possibility of using the ADS questionnaire. The second survey objective was an attempt to translate the results from the ADS scale into the assessment of the perception of people with disabilities in the workplace. To this end, we tried to add a new “work” dimension of statements determining the perception of people with disabilities in the workplace to the ADS scale in an ad hoc manner (see Table 2). These statements were prepared based on the analysis of the results of individual in-depth and group interviews conducted in 2019 on groups of employers, employees with disabilities and persons statutorily supporting the disabled.

Ultimately, two research questions were formulated:

The first research question: Can the WHO measurement tool (the ADS scale), verified by the WHOQOL Group [15] on a group of people with disabilities and those involved in the care of people with disabilities, be used for the whole society? In other words, will the group of factors distinguished in the research of the WHOQOL Group be reflected in a survey carried out on a representative sample of Internet users from different countries?

The second research question: How does the social perception of people with disabilities translate into their perception in the workplace? In other words, which of the social factors identified by the WHOQOL Group [15] have the greatest impact on openness towards such people (a positive perception) in the workplace?

In order to achieve the first objective, we used the analysis of measurement reliability and confirmatory factor analysis (CFA). With the help of CFA, it can also be confirmed whether the structure of factors determining the attitudes of respondents is consistent with the one adopted in the ADS test concept, that is, with the division into four distinguished dimensions.

In order to achieve the second objective, we used a list of items from the proprietary questionnaire concerning issues related to the employment of people with disabilities. As a method of analysis, we used the analysis of measurement reliability and structural equation modelling (SEM) to check whether there is a cause and effect relationship between the factors identified in the ADS test and the assessments of statements concerning the employment of people with disabilities regarding such content as expressed in the items on the ad hoc scale.

## 2. Materials and Methods

### 2.1. Research Method

In the period of September–December 2019, a comparative study was carried out on representative samples of Internet users with the use of computer-assisted web interviews (CAWI). The study covered a group of people from the age of 18 to the official retirement age from eight European countries with different levels of socio-economic development, various existing disability models and systems to support the employment of people with disabilities. Respondents from each country completed the same questionnaire prepared in the national language of a given country (two national languages for Belgium). According to the assumptions, the sample size for each country was to be at least 500 respondents. At the country level, the sample was of random-quota nature and it was representative due to features such as gender, age, education, place of residence and region of residence. Quotas were determined on the basis of the population structure in individual countries and in order to do that, the data from the Eurostat database for 2018 were used. During the implementation of the research, the highest randomness of recruited respondents was ensured by using various ways of reaching them, including Internet panels operating in a given country and mobile applications for telephone/tablet (dynamic sampling). The main research objective was to identify and evaluate various aspects of the perception of people with disabilities. The questions asked to respondents concerned, among others, the general perception of people with disabilities (using the ADS WHOQOL Group scale [15]) and their perception in the workplace, acceptance of various types of disability in the workplace displayed by colleagues, as well as the evaluation of state policy, knowledge of employers about employing people with disabilities, special privileges for people with disabilities in the workplace and social atmosphere in the area of employing people with disabilities. In the article, we focused on the results obtained for questions concerning the general perception of people with disabilities and their perception in the workplace.

### 2.2. Research Sample

During the implementation of the research, 4827 fully completed questionnaires were collected, out of which we selected 4209 respondents constituting the main sample corresponding to the assumed quota structure for each country. For this sample we performed analyses, the results of which are discussed in the article. The characteristics of respondents are presented in Table 3.

The share of respondents from individual countries in the research sample was comparable and amounted to approximately 12.5%. The sample was also balanced in terms of features such as gender (approximately 50% of men and women) and age (approximately one-third of respondents in each of the three distinguished age groups). The highest number of respondents had secondary education, followed by higher education, and the lowest was the share of respondents had received education below the secondary level. The respondents differed in terms of place of residence, both taking into account the size of the city and regions in each country. Most of the respondents were employed, mainly in companies, whereas 1 out of 10 worked in their own business or on a farm. The share of unemployed people looking for a job, retired or benefitting from disability allowances, and learners (from 8.8% to 10.8%), was comparable. The smallest group covered people who were professionally inactive, taking care of the household and not seeking any employment. The respondents who were professionally active (2458 people) answered two additional questions that were intended to clarify information about the nature of the work performed and decision-making regarding the employment of other employees in their workplace. Almost half of the employed respondents are white-collar workers, around 28%-blue-collar workers, whereas the rest of them perform both types of work. The largest group consisted of people who had no influence on the employment of other employees (approximately 40%), whereas the smallest one consisted of respondents with little influence on that (approximately 15%). Among the respondents with a significant impact on employment (about 45%), roughly the same number was obtained for the main decision makers and those making decisions with others, having an equal say in this matter.

People taking part in the survey were asked to specify their experience in dealing with people with disabilities. More than 15% of respondents had extensive experience of this type, resulting from either being a disabled person or taking care of disabled people professionally. Almost one-quarter of respondents did not have any experience in this regard. Other respondents declared that they had contact with people with disabilities in various circumstances—in their families, among friends, in the neighbourhood or in the workplace. At the same time, among those who declared that they had contact with the disabled, the least often mentioned was contact with such people in the workplace (less than 15%), which may also be a manifestation of insufficiency in terms of the needs and possibilities of employment of people with disabilities. On the basis of the respondents’ declarations, we constructed two binary variables describing the experience of disability and presented them in the last row of Table 3. The first variable distinguishes people who declare their own disability and professional care of people with disabilities. It can be assumed that this variable roughly corresponds to the representation of the society studied by the WHOQOL Group [15], for which we adopted the name of involved-in-disability. The second variable distinguishes people declaring lack of experience of disability vs. declaring some experience, and was called experienced-with-disability. It can be assumed that both variables distinguish certain extremes in society—in this research we estimated 15.4% of people involved in disability and 24.1% inexperienced in disability. The comparison of the measurement model in groups makes it possible to answer the question whether the ADS scale is useful in measuring attitudes towards disability in society in general.

### 2.3. Methods of Data Analysis

The analysis of the survey data in relation to the first research question is confirmatory. Firstly, we calculated Cronbach’s alpha reliability coefficients according to classical test theory [18] and compared them with the results of the original publication of Power and Green [15]. In order to verify the validity of the entire tool designed to measure attitudes towards people with disabilities, we used confirmatory factor analysis [19] with four latent variables corresponding to the dimensions of the original ADS scale [15]. In the first stage, all respondents’ answers were taken into account, and then the models estimated in the groups determined by the variables involved-in-disability and experienced-with-disability were compared. The quality of the model was evaluated by testing factor loading values, the significance of coefficients and using the most popular set of goodness-of-fit statistics: CMIN/DF (minimum discrepancy), RMSEA (root mean square error of approximation), GFI (goodness of fit index), AGFI (adjusted goodness of fit index), CFI (comparative fit index) and IFI (incremental fit index). The comparison of fit of the estimated models for the whole sample and with the parameters estimated separately in smaller groups (e.g., people with or without disability experience) was based on the nested models theory [19] (p. 87), in which constraints are placed on the estimated model parameters in order to obtain a model with more degrees of freedom. The difference in fit statistics between them has an asymptotic chi-square distribution with the number of degrees of freedom equal to the difference in degrees of freedom in two models.

In order to evaluate the relationships between the perception of people with disabilities expressed using the ADS WHOQOL Group scale and issues related to their employment, we used structural equation modelling (SEM) which makes it possible to test the whole hypothetical model reflecting the theoretical structure and take into account latent variables. The proposed model contains latent variables included in the ADS scale and a latent variable which is a sufficiently and reliably measured construct related to professional issues. The proposed form of SEM made it possible to check the relationship between the perception dimensions of people with disabilities and their perception in the context of work. It was possible to draw conclusions about the significance, direction and strength of the relationships on the basis of estimated regression weights. The model was also evaluated with the help of goodness-of-fit statistics: CMIN/DF, RMSEA, GFI, AGFI, CFI and IFI. In order to perform statistical calculations and modelling, we used statistical software IBM SPSS 25.0 [20] and Statistica 12.5 [21], including the statistical package SPSS AMOS and SEPATH module in Statistica, and the MS Excel 2013 spreadsheet.

## 3. Results

### 3.1. Measurement Reliability

The results of the measurement reliability analysis performed using the Cronbach’s alpha coefficient are presented in Table 4. The table also contains the values obtained in the original WHOQOL Group research carried out on a sample for intellectual (ID) and physical disability (PD), those obtained on the whole sample, as well as in subgroups of people involved in taking care of the disabled and those who experienced disability on their own.

The obtained values of reliability coefficients are similar in groups. Except for the last factor (prospects), they are lower than the original ones, and below the threshold of 0.7 for reliability adopted in social sciences. However, they exceed the threshold of 0.6, recognised as acceptable for constructs in the development phase [18] (p. 226). Therefore, we gave up the idea of modifying original factors using exploratory factor analysis and decided to carry out validity analysis by means of confirmatory factor analysis.

### 3.2. Measurement Validity by Means of Confirmatory Factor Analysis

**Figure 1:**
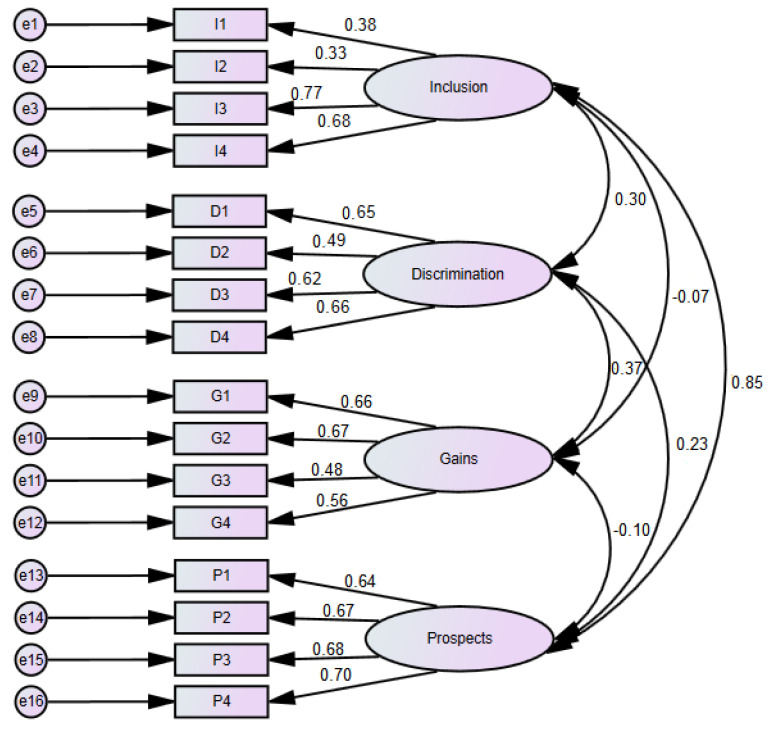
Confirmatory factor analysis model—standardized estimates.

Based on the data collected during the research using the Attitudes to Disability Scale (ADS) of the WHOQOL Group [15], a confirmatory factor analysis was performed. We took into account the responses of all respondents, both of those who had and those who did not have any experience connected with disability. We also used all 16 items of the ADS and specified a 4-factor structure reflecting 4 dimensions: inclusion, discrimination, gains and prospects, as presented by the authors of the scale. It was assumed that the factors are correlated with each other. As an estimation method, we used mainly the maximum likelihood (ML) as the most common one, and for comparison, the asymptotically distribution free (ADF) method with weaker assumptions that are easier to meet in the case of survey data. The results obtained with the use of both methods were very similar. Due to their convergence, the interpretation was based on the results of the ML method. The CFA model, along with ML standardized estimated factor loadings, is shown in Figure 1.

Table 5 presents standardized values of factor loadings. All items achieved high statistical significance (*p* < 0.001), which indicates that the model does not reject the assumed construct. All factor loadings are positive and their standardized values range from 0.332 to 0.767. Loadings lower than the limit of 0.5 occur with variables I1 (0.376), I2 (0.332), D2 (0.486) and G3 (0.482). The two first loadings are well below the limit, but two latter are very close to this limit. For the prospects latent variable, all factor loadings exceed the value of 0.6. In the case of the inclusion latent variable, two low loadings (0.376 and 0.332) and two high ones (0.767 and 0.681) were noted. Between the inclusion and prospects variables it is possible to notice a strong positive correlation (0.852). Although we expected negative correlations of the gains factor with the others due to the reverse formulation of the items, the correlation with the discrimination factor appeared to be positive.

The literature on the subject recommends using different measures in order to assess the quality of the model [22]. They enable checking the degree of fit between the model specified by the researcher and sample data [23]. Table 6 includes commonly used goodness-of-fit statistics. Chi-square statistic and the measure CMIN/DF associated with it are high (1606.0 and 16.387, respectively), whereas the *p*-value is below the threshold. However, the usefulness of these indicators as tools for assessing model fit is often questioned. Although the desirable situation is an insignificant chi-square statistic with a *p*-value higher than 0.05, in practice it rarely happens, especially in the case of a large sample [24,25]. Barrett [26] emphasizes that this is the only exact-fit test for SEM, but that it is problematic due to the very high sensitivity to discrepancies in relation to the values expected in the case of a large sample. Bentler and Bonett [27] claim that the chi-square statistic is a function of the sample size, and with higher *n,* the probability of rejecting the null hypothesis about the lack of differences increases, even if the estimated model is slightly incorrect. Hair et al. [28] indicate that for *n* > 250 and the number of observable variables higher than 12, one should expect a significant *p*-value. The considered CFA model was estimated on the basis of a sample of *n* = 4209 and 16 observable variables, so the values related to the chi-square statistic should be treated with caution and cannot constitute the sole basis for assessment. Due to the indicated limitations of the chi-square statistic in model testing, various descriptive measures are used to assess the fit, including RMSEA, GFI, AGFI, CFI, IFI [28]. Root mean square error of approximation (RMSEA) is a measure of overall model fit. RMSEA is equal to 0.06, which means a reasonable error of approximation, i.e., lower than the threshold of 0.08 [29]. Goodness of fit index (GFI) and adjusted goodness of fit index (AGFI) are absolute measures of fit, with AGFI containing correction for degrees of freedom. Both assume values lower than one, whereas high values indicate a good fit of the model. Cut-off point is a matter of discussion—most often postulated values are greater than 0.9 [30] or 0.95 [31]. In the considered model, GFI = 0.95 and AGFI = 0.930, which suggests a satisfactory fit. Comparative fit index (CFI) and incremental fit index (IFI) belong to the group of relative fit indices comparing the analysed model with the independence model. Both are normalized in the range from 0 to 1, and values close to 1 are interpreted as a very good fit. For CFI, values higher than 0.95 are usually recommended [32], although the threshold of 0.9 [33] was suggested in previous studies. For the specified model CFI = 0.903 and IFI = 0.903, which is slightly above the acceptance limit.

Although the fit of the model can be considered acceptable, it is necessary to emphasise the weaknesses revealed in the measurement one. First of all, in this study, factor 1 originally identified as inclusion breaks down into two factors. Items I1 and I2 in exploratory factor analysis would form a separate factor in relation to items I3 and I4, which would combine with factor 4—prospects (high correlation between them). The original results do not include the correlation between the factors in the model, but the published low values of item–total correlation occur only for factors 2 and 3, which corresponds to a stronger association of factors 1 and 4. Therefore, it was decided to leave the original ADS Scale for further analysis without modification. On the basis of factor loadings, we also calculated composite reliability coefficients which took, as usual, slightly higher values than Cronbach’s alpha, but did not lead to conclusions other than the alpha coefficient.

### 3.3. Nested Models of CFA in Groups

The acceptance of the measurement model in the whole sample can be supplemented by the analysis of models estimated separately in groups designated by the involved-in-disability and experienced-with-disability variables. According to the theory of nested models, we compared the richer model containing separate parameters in groups with models that had imposed constraints. The constraints were imposed in two ways: the equality of parameters in both groups without imposing their values, and the equality of parameters in both groups obtained in the whole group, treated as fixed parameters.

The values of the parameters obtained in the groups were similar to those obtained in the entire population treated as constants. While comparing the fit of models assuming the equality of parameters in groups, it can be noticed that there is no significant difference in the fit of the model with this constraint in the case of the experience-with-disability variable (see Table 7). This means that the measurement model has the same form regardless of declaring or not declaring experience of disability. A significant difference in fit was observed in the models for the involvement-in-disability variable. However, the similarity of parameter values to those obtained in the overall sample should be considered. This leads us to the conclusion that a measurement of attitudes towards disability using the scale developed by WHOQOL Group can be adopted for the whole society.

### 3.4. Measurement of Perception of Disability in Work

While preparing our own scale of measurement, we expected a one-factor structure. However, the exploratory factor analysis (EFA) shows a clear two-factor structure, so the initial concept was too general and the scale requires refinement. Factor loadings obtained by principal components with rotation Varimax are presented in Table 8.

Comparing factor loadings with the content of items from Table 2, it is possible to notice that the factors got separated in terms of positive (W1 and W4) or negative (W2 and W3) approaches towards disability in the workplace and evaluating people with disabilities (W2 and W3), or assessing the situation of a company employing people with disabilities (W1 and W4). Cronbach’s alpha reliability coefficients took the value of 0.481 for items W1 + W4, which means that they do not form a reliable summative measurement scale, and variables W1 and W4 should be treated separately in the analysis, which was not done in this article. We focused on using items W2 + W3 as a scale for measuring the perception of employing people with disabilities, for which Cronbach’s alpha coefficient of 0.621 was obtained, which means acceptable reliability for constructs in the development phase [18] (p. 226). These items focus on the core of perception of people with disabilities and were used in the next step of the research in order to assess which aspects of the general attitude towards disability measured by the ADS scale affect the specific perception of disability in the workplace.

### 3.5. Results for the Structural Equation Model

**Figure 2:**
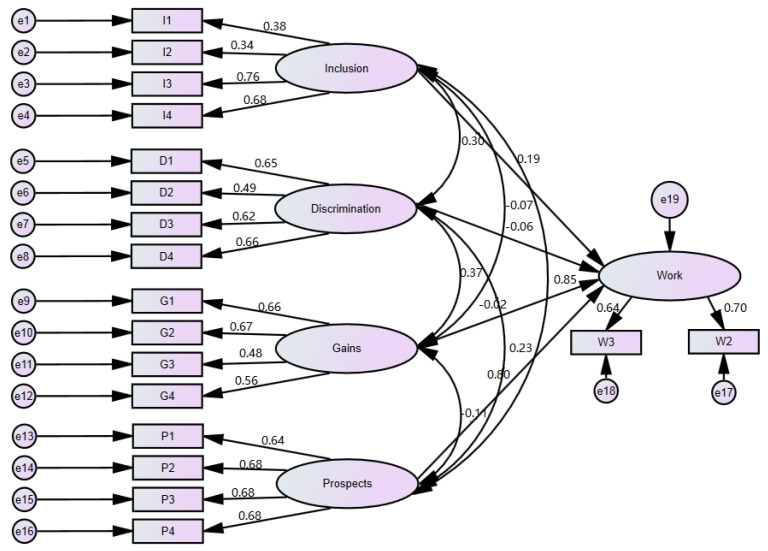
Structural equation model—standardized estimates.

The hypothetical structural equation model describing the relationships between variables is shown in Figure 2. It contains the measurement part discussed and verified in the previous part of the article (cf. 3.1), and examines the relationship between the attitude towards people with disabilities and their assessment in the context of professional work. According to the diagram, the dimensions of perception such as inclusion, discrimination, gains and prospects affect the evaluation of people in the workplace. Although initially 4 variables were proposed for the latent work variable in the questionnaire, on the basis of EFA and reliability analysis, we left only two in the model—W2 and W3. The standardized estimates corresponding to them are relatively high—0.702 and 0.642, respectively (ADF estimates 0.696 and 0.622, respectively).

Regression weights for predictors of the work variable are illustrated in Table 9. Coefficients for inclusion and prospects are positive, whereas they are negative for discrimination and gains. Three predictors—inclusion, discrimination and prospects—have a significant impact on the perception of people with disabilities as employees, while the impact of gains is insignificant. The standardized estimates indicate that prospects is the strongest factor (0.800 value). For the other predictors, the values are definitely lower: −0.193 for inclusion and −0.058 for discrimination. The low value of standardized regression weight for the discrimination variable suggests that the impact of this variable is low.

Table 10 presents goodness-of-fit statistics for the analysed structural equation model. The chi-square statistic is 1763.6 (*p* = 0.000), and the CMIN/DF measure is 14.109. Such values can be expected due to the sample size and the number of observable variables in the model. RMSEA = 0.056 is on an acceptable level. The absolute fit measures GFI = 0.951 and AGFI = 0.932 take acceptable values. Relative measures CFI = 0.915 and IFI = 0.916 are quite close to 1, but their values do not exceed the 0.95 threshold indicated by some authors. The presented quality measures allow the acceptance of the proposed model.

## 4. Discussion

The final version of the ADS scale WHOQOL Group was developed after numerous tests and studies [15]. It contained four subscales, each in the final version consisting of four items. Such a division was mainly the result of focus group interviews carried out in a group of experts in the field of disability, among people with physical and intellectual disabilities as well as their caregivers. One can also take into consideration the results of the pilot study covering 38 items, which were analysed using the confirmatory factor analysis (CFA) and item response theory (IRT). The results obtained by the authors from the WHOQOL Group confirmed the appropriate adjustment of the created tool for studying attitudes towards people with disabilities. It should be noted, however, that the tool was tested only on people dealing with disability issues, that is, people suffering from disabilities and people professionally or privately supporting this target group.

The subsequent studies focused either on analogous groups or on groups of people who currently take care of people with disabilities or will be involved in that in the future—physiotherapy students [35] and social workers [36]. An attempt was also made to assess the ADS scale in a broader context, taking into account the whole society [37]. However, the assessments did not concern the direct opinion of various social groups, but the attitude of society towards people with disabilities. Still, the research continued to focus on the opinions of an analogous target group of people with disabilities and their caregivers.

We attempted to use the ADS scale WHOQOL Group to gather opinions of the whole society about people with disabilities and verify its adequacy in such a research group. The obtained results allow the acceptance of the ADS scale measurement model, as well as the recognition that it is adequate for use in the wider group of a whole society. However, it should be noted that not all measures are satisfactory. The first two items from the inclusion factor have low loadings, which means that they do not correlate properly with the other items. The exploratory factor analysis showed that they should be a separate group, while items number 3 and 4 should be included in factor number 4 (prospects). Comparing these results with the detailed analysis of Power and Green research [15], it can be noted that they do not differ from the expected ones. The same two items were qualified by the authors of the scale in the pilot study for the same factor as the statements from the prospects factor, and the results of the final scale reliability study indicated that in the IRT analysis, they are characterized by a disordered threshold (at least in the case of people with mobility impairments). Therefore, the modification of the ADS scale by the WHO should be considered for the needs of future research, provided that other researchers obtain similar results in independent studies. It is also worth trying to use exploratory factor analysis (EFA) in order to isolate groups of factors, and not just confirm the compliance of the adopted concept with the test results.

In this article, we also attempted to assess the relationship between the general perception of disability and the perception of employing people with disabilities (lack of adequate comparative research). To this end, on the basis of interviews, we created our own ad hoc scale which consisted of four items—two negative and two positive; two assessing the attitudes and capabilities of people with disabilities, and the other two relating to the impact of employing people with disabilities on the situation of the company. This scale made it possible to measure the perception of disability in the workplace and identify aspects which have influence on it. Of the four distinguished dimensions of social perception of disability, the assessment of prospects has the greatest impact on relationships at work. The more the respondents agreed with the opinion that people with disabilities can think in a similar way as people without disabilities, the more often they accepted the opinion that such people are willing to work and are not characterised by lower work performance. A similar situation occurred in the case of the inclusion dimension. Among the factors of general attitudes towards disability, there is a lack of significant impact of the only positively formulated gains factor on perception in the workplace. The impact of the discrimination factor is weak and negative, that is, the more often one notices that people with disabilities are socially discriminated, the better they are perceived in the labour market.

It is worth noting that in the studies of Power et al., they compared the results of research on the perception of people with disabilities, taking into account demographic and geographical features of respondents [15,16]. There were no differences in the case of e.g., gender, but it was possible to notice significant discrepancies depending on the place where the research was carried out. Therefore, when planning further research, it is worth focusing on differences between countries and comparing the level of measured variables rather than their dependencies. This will make it possible to determine the conditions and potential path of the transition of less-developed societies and economies to provide stronger support to people with disabilities in finding employment. In further research, one should also improve the scale of measuring the perception of disability in the workplace, expanding both aspects taken into consideration in previous studies performed by the authors of the article—the attitude towards the disabled and the perception of the possibilities of companies employing people with disabilities. The results obtained in subjective studies should also be compared with objective data from the Labour Force Study in the field of actual employment of people with disabilities in specific European countries (see EU methodological user guide [38] and results for Poland [39]).

## 5. Conclusions

Although it was expected that the measurement reliability and the fit of the ADS WHOQOL Group [15] scale measurement model would be at a higher level, it can be considered high enough to be used to measure attitudes towards people with disabilities in various aspects. Results similar to the entire sample were obtained in groups designated on the basis of respondents’ declarations regarding experience of disability. Factors of the ADS scale were included in the SEM model as predictors of the perception of employing people with disabilities. The most significant influence on the perception of disability at work was the attitude towards the perspective of people with disabilities and their inclusion in society. If this relationship is true, in order to improve the situation of people with disabilities in the labour market, one needs to address the stereotypes associated with these two factors.

## Figures and Tables

**Table 1:** Attitudes to Disability Scale (ADS) developed by WHOQOL Group [15].

Area/Factor	Item	Symbol
Inclusion	1. People with a disability find it harder than others to make new friends	I1
	2. People with a disability have problems getting involved in society	I2
	3. People with a disability are a burden on society	I3
	4. People with a disability are a burden on their family	I4
Discrimination	5. People often make fun of disabilities	D1
	6. People with a disability are easier to take advantage of (exploit or treat badly) compared with other people	D2
	7. People tend to become impatient with those with a disability	D3
	8. People tend to treat those with a disability as if they have no feelings	D4
Gains	9. Having a disability can make someone a stronger person	G1
	10. Having a disability can make someone a wiser person	G2
	11. Some people achieve more because of their disability (e.g., they are more successful)	G3
	12. People with a disability are more determined than others to reach their goals	G4
Prospects	13. Sex should not be discussed with people with disabilities	P1
	14. People should not expect too much from those with a disability	P2
	15. People with a disability should not be optimistic (hopeful) about their future	P3
	16. People with a disability have less to look forward to than others	P4

**Table 2:** Proposed statements on the perception of people with disabilities in the workplace.

Area	Statement	Symbol
Work	17. Employing people with disabilities improves company’s image	W1
	18. People with disabilities do not want to work, they do not look for a job	W2
	19. People with disabilities work less efficiently than people without any disabilities	W3
	20. The limitations resulting from disabilities can be effectively compensated by a suitable workplace or its equipment	W4

**Table 3:** Main research sample (*n* = 4209)—social, demographic and professional features.

Feature	Feature Categories	Percentage of Respondents (%)
Country	Belgium	12.4
	Bulgaria	12.5
	Germany	12.7
	Greece	12.3
	Poland	12.5
	Spain	12.4
	Sweden	12.4
	United Kingdom	12.7
Sex	Female	49.9
	Male	50.1
Age	18–34 years old	32.4
	35–49 years old	33.6
	50–65 years old	34
Education	Below secondary (e.g., elementary/middle school/vocational) or no education	21.7
	Secondary with or without a high school diploma/post-secondary	43.7
	Higher	34.6
Size of the place of residence	Countryside	19.6
	City up to 50 k residents	30.7
	City from 50 k to 200 k residents	22.2
	City from 201 k to 500 k residents	11.6
	City over 500 k residents	15.9
Main current professional status	Learner/student	8.8
	Employed, work for someone	47.8
	Work in own company, farm	10.6
	Retirement/pension	9
	Unemployed, seeking work	10.8
	Not working, housekeeping, not looking for work	6.9
	Other	6
Kind of work ^1^	Blue-collar worker	27.9
	White-collar worker	48.1
	Both types of work	24
Decision maker in regard to employing people ^1^	Main decision maker	21.8
	Make decisions together with the other people	23.2
	Little influence on the decision	14.7
	Does not make decisions on employing other people	40.3
Declaration of experience in disability	Involved-in-Disability:	
	Yes	15.4
	No	84.6
	Experienced-with-Disability:	
	Yes	75.9
	No	24.1

^1^ Only employees or working in own company, farm (*n* = 2458).

**Table 4:** Comparison of Cronbach’s alpha reliability coefficients obtained in the original [15] and current research (ID—intellectual disability, PD—physical disability).

ADS Factor	Original Alpha Values		Received Alpha Values				
	ID	PD	Whole Sample	Involved-in-Disability		Experienced-with-Disability	
				Yes	No	Yes	No
Inclusion	0.669	0.714	0.626	0.630	0.624	0.632	0.607
Discrimination	0.737	0.754	0.692	0.684	0.690	0.691	0.690
Gains	0.789	0.760	0.683	0.626	0.693	0.668	0.723
Prospects	0.720	0.790	0.766	0.791	0.761	0.770	0.750

**Table 5:** Four-factor model: parameter estimates and significance (ML—maximum likelihood, ADF—asymptotically distribution free).

Latent Variable	Item	ML Standardized Factor Loading or Correlation	ADF Standardized Factor Loading or Correlation
1. Inclusion	I1	0.376 ***	0.374 ***
	I2	0.332 ***	0.318 ***
	I3	0.767 ***	0.786 ***
	I4	0.681 ***	0.660 ***
2. Discrimination	D1	0.650 ***	0.593 ***
	D2	0.486 ***	0.421 ***
	D3	0.618 ***	0.621 ***
	D4	0.664 ***	0.621 ***
3. Gains	G1	0.663 ***	0.696 ***
	G2	0.668 ***	0.611 ***
	G3	0.482 ***	0.473 ***
	G4	0.564 ***	0.477 ***
4. Prospects	P1	0.639 ***	0.608 ***
	P2	0.668 ***	0.648 ***
	P3	0.677 ***	0.663 ***
	P4	0.698 ***	0.679 ***
Correlations between factors	r(1,2)	0.300 ***	0.275 ***
	r(1,3)	−0.073 ***	−0.191 ***
	r(1,4)	0.852 ***	0.819 ***
	r(2,3)	0.373 ***	0.233 ***
	r(2,4)	0.230 ***	0.183 ***
	r(3,4)	−0.105 ***	−0.265 ***

*** *p* < 0.001.

**Table 6:** Characteristics and goodness-of-fit statistics for the four-factor model.

Measure	ML Score	ADF Score
Number of parameters	38	38
Chi square	1606.0	1107.4
df	98	98
*p*	<0.0001	<0.0001
CMIN/DF (minimum discrepancy)	16.387	11.300
RMSEA (root mean square error of approximation)	0.060	0.049
GFI (goodness of fit index)	0.950	0.934
AGFI (adjusted goodness of fit index)	0.930	0.908
CFI (comparative fit index)	0.903	0.729 ^1^
IFI (incremental fit index)	0.903	0.730 ^1^

^1^ Comparative and incremental fit indices of reject models estimated with the ADF method [34] (pp. 89–96).

**Table 7:** Characteristics and goodness-of-fit statistics for the four-factor model.

Binary Variable, Constraint	Difference of Chi-Square Statistics	df	*p*
Involved-in-disability, equal parameters in groups	43.045	22	0.005
Involved-in-disability, parameters equal to the whole sample	43.458	76	0.999
Experienced-with-disability, equal parameters in groups	25.704	22	0.265
Experienced-with-disability, parameters equal to the whole sample	25.856	76	1

**Table 8:** Factor loadings of perception disability in work items.

Variables	Factor 1	Factor 2
W1	0.005	0.824
W2	0.839	−0.125
W3	0.855	−0.015
W4	−0.141	0.791

**Table 9:** Regression weights: parameter estimates, standard errors and critical ratios.

Relation	ML Standardized Estimate	ADF Standardized Estimate
Work <--- Inclusion	0.193 ***	0.135 **
Work <--- Discrimination	−0.058 **	−0.077 *
Work <--- Gains	−0.019	−0.001
Work <--- Prospects	0.800 ***	0.848 ***

* *p* < 0.05, ** *p* < 0.01, *** *p* < 0.001.

**Table 10:** Characteristics and goodness-of-fit statistics for the structural equation model.

Measure	ML Score	ADF Score
Number of parameters	47	47
Chi square	1763.6	1233.0
df	125	125
*p*	0.000	0.000
CMIN/DF (minimum discrepancy)	14.109	9.864
RMSEA (root mean square error of approximation)	0.056	0.046
GFI (goodness of fit index)	0.951	0.930
AGFI (adjusted goodness of fit index)	0.932	0.904
CFI (comparative fit index)	0.915	0.725 ^1^
IFI (incremental fit index)	0.916	0.727 ^1^

^1^ Comparative and incremental fit indices of reject models estimated with the ADF method [34] (pp. 89–96).
